# A Properly Balanced Reduction Diet and/or Supplementation Solve the Problem with the Deficiency of These Vitamins Soluble in Water in Patients with PCOS

**DOI:** 10.3390/nu13030746

**Published:** 2021-02-26

**Authors:** Małgorzata Szczuko, Iwona Szydłowska, Jolanta Nawrocka-Rutkowska

**Affiliations:** 1 Department of Human Nutrition and Metabolomics, Pomeranian Medical University in Szczecin, Broniewskiego 24, 71-460 Szczecin, Poland; 2 Department of Gynecology, Endocrinology and Gynecological Oncology, Pomeranian Medical University in Szczecin, Unii Lubelskiej 1, 71-256 Szczecin, Poland; iwona.szydlowska@pum.edu.pl (I.S.); jolanta.nawrocka@pum.edu.pl (J.N.-R.)

**Keywords:** PCOS, vitamins, thiamine, folates, niacin, ascorbic acid

## Abstract

Polycystic ovary syndrome (PCOS) is an increasingly common problem for women in the reproductive age throughout the entire world. A reduction diet with a low glycaemic index (GI) has proved to support the treatment of PCOS. The aim of the study was to analyse the influence of the diet on the level of vitamins soluble in water. The study included 55 women, 40 of which suffered from PCOS (identified by means of the Rotterdam Criteria) and 15 healthy women of the Caucasian race. The level of vitamins before and after the dietary intervention was measured. The diet was a reduction diet with a reduced glycaemic index (GI). Biochemical analyses were made on the basis of liquid chromatography—Infinity 1260 Binary liquid chromatography (LC) Agilent Technology. The level of vitamins in the serum was analysed together with the consumption before and after the dietary intervention. A higher level of vitamin C in the plasma was observed before and after the dietary intervention in the PCOS group in comparison to the control group despite the lower intake of this vitamin in the PCOS group. The remaining vitamins were at a comparable or lower level (B1, B3, B5, B6 and B12). After the dietary intervention, only B1 and B9 were at a clearly lower level (a trend of *p* = 0.093 and *p* = 0.085). A properly balanced reduction diet with reduced GI improves the supply of vitamins in women with PCOS. An additional recommendation should be the additional supplementation of B1, niacinamide and the combination of folates with inositol. The level of vitamin C in the plasma may not be a good marker of its supply in the PCOS group.

## 1. Introduction

Polycystic ovary syndrome (PCOS) is diagnosed on the basis of at least two out of three Rotterdam Criteria from 2003 [1]:

- hyperandrogenism,
- or its clinical manifestations, such as androgenic baldness, hirsutism,
- menstruation and ovulation disorders,
- enlarged (ovary volume > 10 cm^3^) or polycystic ovaries (at least 12 follicles) visible in an ultrasound image.

When it comes to metabolism, PCOS is characterised by disorders in carbohydrate metabolism, which is accompanied by insulin resistance [2]. Furthermore, the fat build-up of internal organs, particularly, the presence of NAFLD, is the domain of women [3,4]. A study published in 2017 proved that the implementation of a reduction diet combined with physical activity had an influence on the reduction in body mass in patients and on the improvement of biochemical parameters, mainly of the lipid profile, which is often disturbed [5]. Therefore, it seems that PCOS is a syndrome that includes numerous disorders that originate in metabolic defects leading to the development of obesity. The presence of the symptoms depends on the effective functioning of the entire organism, while the dysfunction of ovaries is a consequence of metabolic disorders. Women with PCOS are also in the risk group of developing type 2 diabetes, which makes more sensitive to insulin. This is why metformin is often used in the treatment of PCOS. Unfortunately, this medicine has an influence on the reduction in vitamin B_12_ levels after just a few months of intake and is accompanied by an increase in the concentration of homocysteine [6]. Moreover, the inability to get pregnant and random stillbirth in women with PCOS may also be a consequence of the clinical deficiency of B12 [7]. In addition, in patients with hyperhomocysteinemia, stillbirth was observed more frequently than in women with correct homocysteine concentration [8].

The authors have a hypothesis that vitamins soluble in water that have antioxidant properties and participate in metabolic transformations as regulators may be supplemented together with a reduction diet, thus being beneficial in the treatment of PCOS. In the available literature, we did not find information on the influence of a balanced reduction diet on the supplementation of nutritional deficiencies with regards to vitamins soluble in water in women with PCOS. We were interested whether the supply combined with a properly balanced reduction diet proves sufficient to negate the differences with the control group, whether this type of diet should also additionally include supplementation and if so—what vitamins should be selected. We were interested whether the supply with an appropriately balanced reduction diet is enough to eliminate the difference in comparison to the control group or whether this type of diet should also include supplementation with specific vitamins (which ones?).

We decided to check whether the reduction diet with a low glycaemic index (GI), but rich in vitamins can improve the status of vitamins soluble in water in women with PCOS. To do this, we compared the results to those of women with PCOS who did not decide to change the diet, as well as to healthy women with excluded PCOS.

## 2. Material and Methods

The study was approved by the Bioethics Committee of the Pomeranian Medical University in Szczecin and was conducted in accordance with the provisions of the Declaration of Helsinki, institutional policy, and national law. All study participants consciously expressed their consent to participate in the study.

### 2.1. Study Group

In total, 55 women of the Caucasian race participated in the study—40 patients aged 32.52 ± 7.12 years with polycystic ovary syndrome (PCOS-I) diagnosed on the basis of the Rotterdam criteria: 2 out of 3 of the following criteria—rare ovulations or lack of ovulations and/or biochemical symptoms of hyperandrogenism and/or image of polycystic ovaries in USG (Ultrasound Voluson 730, GE, Switzerland). The control group (CG) consisted of 15 women aged 31.23 ± 6.3 years and the correct BMI of 22.1 ± 1.5 without PCOS. All participants of the study were subjected to the measurements of anthropometric bioelectrical impedance (Akern, BIA-101, Firenze, Italy).

**Figure 1:**
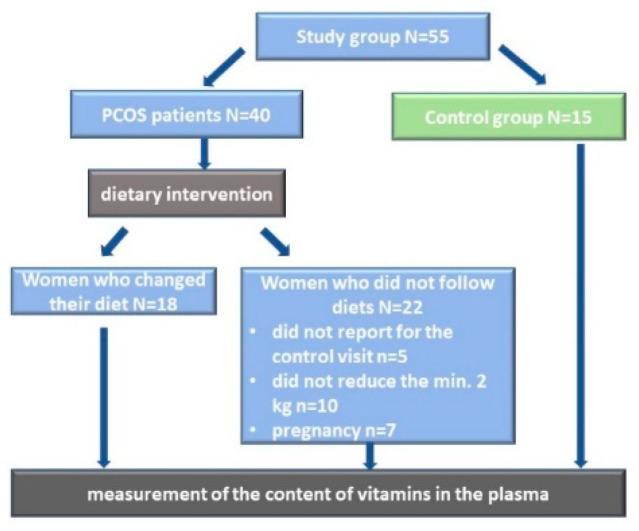
Study design.

Only 18 women with polycystic ovary syndrome who followed the recommendations and diet were qualified for stage 2 of the study (PCOS-II). The verification was conducted on the basis of a nutritional interview and body mass reduction—a minimum of 2 kg in 3 months. Lastly, the measurement of the content of vitamins in the plasma was conducted and compared between the groups in accordance with Figure 1. All of the women were in the childbearing age and their anthropometric parameters are presented in Table 1.

### 2.2. Quantitative Dietary Assessment

The following methods were used to gather data on product consumption: food diary referring to the last 3 days at the start of the study. The focus was on a one-day food record from the last 24-h dietary interview when the patient returned for a control visit. The data collected from the diaries and interviews included the following: quantity, way of preparation, the time of consumption of each meal and the ingredients that were used. The menus were taken on Thursday and Friday as well as on Saturday or Sunday. Using computer software Dieta 6D (National Food and Nutrition Institute, Warsaw, Poland), we analysed a total of 162 dietary menus of women suffering from PCOS.

### 2.3. Dietary Intervention

Recommendations pertaining to the change of lifestyle and a 7-day menu were provided to each female participant [9,10]. The caloricity of the diet was reduced by 600 kcal with reference to the daily caloric needs. Moreover, 5 meals per day were included in the diet, and the products were given in grams. Sources of carbohydrates (5 portions per day) included: brown rice, oatmeal, coarse-grained groats, wholegrain rye bread or graham bread and—sporadically—potatoes as well as wholemeal pasta featuring lowered glycaemic indexes. The diets recommended the following products as sources of proteins: lean meat without skin (turkey or chicken), fish (sole, salmon and tuna), eggs, semi-skimmed milk (fat content: 2%), dairy products (quark and natural yoghurt) and legumes (soy, red lentils, beans or peas). Fat sources (2 portions per day) included—nuts and seeds (pumpkin seeds, almonds, sunflower seeds, sesame seeds, and chia seeds), oily fruits, e.g., avocado, and raw cold oils (e.g., linseed oil, olive oil, and rapeseed oil). From October to April, it is recommended to consume cod liver oil as Poland is situated in a temperate, warm transitional climate as people here often have problems with vitamin D deficiency.

Low GI fruits and vegetables were also included in the menus with the aim of supplementing the diet with minerals and vitamins. The recommended methods of food preparation were braising, roasting, cooking in water and steaming. Furthermore, every participant was advised to drink about 2 L of fluids every day, especially water and herbal infusions. The final recommendation was to increase physical activity to a minimum of 3 h a week.

### 2.4. Reagents for Biochemical Analyses

Reagents (NaHCO3, NaOH, HK_2_PO_4_, methanol and acetonitrile) of the highest HPLC quality were purchased from Sigma Aldrich (St. Louis, MO, USA). Millipore water (Millipore, Billerica, MA, USA) was used to prepare the buffers. Vitamins were isolated using amber-coloured Eppendorf tubes. To secure vitamins against photo-oxidation, the samples were isolated in a dark room in amber Eppendorf tubes. After the collection of 400 µL of blood plasma, an equal amount of acetonitrile was added together with 100 µL of internal standard (100 ng/mL theobromine). The reagents were mixed for 2 min and then centrifuged for 15 min at 4000 rpm. The formed supernatant was then transferred to new tubes in order to evaporate the acetonitrile. The water phase was moved to solid phase extraction columns that included a C-18 silica cartridge (Thermo Scientific, Waltham, MA, USA), which was activated earlier by means of 1 mL of methanol and 1 mL of clear water. The contents of the columns were subjected to elution using 85% methanol with 1.5 mL water. The formed solution was vacuum dried and—directly before HPLC analysis—was diluted in 100 µL buffer, 25 mM HK_2_PO_4_ [11,12].

### 2.5. The Analysis of Vitamins Soluble in Water with HPLC

HPLC Infinity 1260 Binary LC (Agilent Technologies, Waldbronn, Germany) was used to conduct the analysis. Subsequently, vitamins were separated using the gradient method with 25 mM HK_2_PO_4_ buffer with the pH of 7.0 and 100% methanol buffer. A and B buffer proportions for the times 0.0, 2.5 and 16 min were 97%:3%, while for 7.2 and 14 min they were 70%:30%. The separation of vitamins was conducted using BDB Hypersil C-18 (Thermo Scientific) column at 35 °C. The buffers moved through the column at the rate of 0.9 mL/min, and the injection volume was 10 µL [13]. The identification of specific vitamins in the studied samples was conducted on the basis of the observation of retention times of standard peaks. The data were analysed using ChemStation taking into account standard curves (ascorbic acid-C, thiamine-B1, riboflavin-B2, nicotinic acid-B3, calcium pantothenate-B5, pyridoxine-B6, biotin-B7, folic acid-B9, cyanocobalamine-B12, niacinamide-PP) with a correction for the internal standard (theobromine with the concentration of 100 mg/mL) [14].

### 2.6. Statistical Analysis

Statistica 12.0 (StatSoft, Cracow, Poland) was used to analyse the results. The average values (Avg) and standard deviation (SD) were calculated. As the distribution in most cases deviated from normal (Shapiro–Wilk test), non-parametric tests were used: the Mann–Whitney test for group comparisons (PCOS and CG) in which *p* < 0.05 was considered statistically significant.

## 3. Results

The average consumption of vitamins in the analysed diets in the PCOS-I group and—especially—in the PCOS-II group significantly differed in comparison to the control group (Table 2). The participants of the PCOS-I group consumed less vitamins in comparison to the control group, but statistical significance was observed only in terms of vitamin C and folates. The PCOS-II group consumed statistically significantly higher amounts of all of the analysed vitamins in comparison to PCOS-I. When comparing the consumption of vitamins in PCOS-II with the control group, statistically significant differences or trends were also observed in terms of the higher consumption of vitamins, except for cobalamin (Table 2).

When investigating the supply of vitamins soluble in water, we based our analysis on their concentration in blood plasma. After the dietary intervention, the concentration of some of the vitamins changed significantly (Figure S1A–C; Table 3). Most vitamin levels in the analysed cases were significantly different between PCOS-I and the control group (CG) (Figure S1B; Table 3). This was true for vitamin C, thiamine, B3, B5, pyridoxine, folates and cobalamin—in the last case, a trend was observed. However, the concentrations of vitamin C and B3 were lower in the control group. After the introduction of a balanced reduction diet, vitamin C and B3 levels in the PCOS-II group still remained significantly lower than in CG (which was surprising), but they did improve insignificantly (Figure S1C; Table 3). Furthermore, the average level of the remaining vitamins was increased in comparison to PCOS-I, but the results were not statistically significant (Table 3).

## 4. Discussion

When analysing vitamin levels in the plasma, we took into account their supply and the needs of the human body depending on age, physical activity and accompanying illnesses associated with increased needs. The polycystic ovary syndrome is associated with the presence of a chronic inflammation and increased oxidative stress. This is why, the supply of antioxidants, including vitamins, is particularly important. Vitamins that have antioxidant properties include ascorbic acid, the fastest reacting antioxidant, as well as vitamin E, carotenoids and flavonoids [15]. Ascorbic acid (AA) is present in high concentrations in the pituitary gland. Therefore, it can play a significant role in the secretion of the anterior pituitary hormones, including follicle stimulating hormone (FSH), luteinizing hormone (LH) and prolactin (PRL) [16]. Moreover, it has been determined that AA deficiency (caused by low consumption) increases insulin resistance, which accompanies women with PCOS. This is why, the diet offered to women with PCOS was rich in this constituent (the average consumption of 230 mg/day/person). In our opinion, the observed higher concentration of vitamin C in the plasma of women with PCOS both before and after the dietary intervention is associated with the organism’s response to oxidative stress and the competition with glucose for the joint transporter GLUT1 and GLUT3 to cell interior [17]. Due to the fact that vitamin C level in the plasma—regardless of its supply with the diet—remained at the same level (PCOS-I and PCOS-II), it seems that the measurement of AA levels in the plasma of these patients cannot serve to measure the supply of this vitamin to the organism. The problem was already described earlier by the authors in 2019 [14]. Additionally, in the same study group, an increased level of several eicosanoids was observed after the introduction of a reduction diet with reduced GI. The same authors explained the mechanism as activation/amplification of repair processes [18].

Group B vitamins belong to the category of vitamins whose main function is regulation, which means that they participate in important processes and reactions located within tissues and cells. Due to the possibility of dysbiosis in the course of PCOS, it seems very important to supplement the supply of these vitamins [14,19]. In the study, in the case of most B vitamins, the increase in their supply with the diet lead to the expected result in the form of their increased level in the plasma of women with PCOS. This effect was not observed for vitamin B3, and the levels of B2 and thiamine were not as satisfactory as in the case of the remaining vitamins. This is why the authors decided to analyse the cause of this effect. It has been documented that the insufficient supply of vitamin B_3_ is associated with the development of inflammatory diseases [20]. Some examples of this type of disorders that accompany the pathogenesis of PCOS include insulin resistance and lipid disorders that promote atherosclerosis, as well as the increased risk of cardiovascular diseases [21,22]. Therefore, the delayed removal of fat from circulation in women with PCOS is another factor supporting the occurrence of this syndrome. It has been shown that nicotinic acid therapy reduces the frequency of occurrence of stroke and myocardial infarction and alleviates coronary artery revascularisation in patients suffering from the metabolic syndrome [23]. Niacin is important for the increase in HDL, the decrease in plasma TG and low-density lipoprotein (LDL) [24]. Radmila Lyubarova et al. (over 3000 patients) state that extended-release niacin (ERN) is associated with the decrease in the activity of lipoprotein-associated phospholipase A_2_ (LpPLA_2_) and hence, with the risk of cardiovascular (CV) events [25]. Furthermore, nicotinamide and its metabolite N1-Methylnicotinamide (MNAM) alleviate endocrine and metabolic abnormalities in ovarian and fat tissues in the rat model of PCOS [26]. It has been observed that MNAM production is significantly higher in the cumulus cells of PCOS patients. It has also been demonstrated that its administration in the rat model of PCOS helped solve the problem of hyperandrogenism and ovarian adenosine monophosphate-activated protein kinase (AMPK). This is possible via aldehyde oxidase 1 (AOX1), which is a detoxifying enzyme that metabolises MNAM through the transient elevation of ROS [27]. Nicotinamide is a direct precursor used in the synthesis of NAD+ and NADP+, which are important coenzymes of redox reactions. Data shows that nicotinic acid is not formed from nicotinamide in the human body. On the contrary, nicotinic acid has to be transformed into nicotinamide [28]. Therefore, in our study, due to the protective effect with reference to vascular endothelium and the antithrombotic potential, it seems that the introduction of a balanced reduction diet rich in antioxidants lead to the activation of repair mechanisms resulting in the observation of a reduction in the level of nicotinic acid in the plasma of women with PCOS. Therefore, it seems that additional supplementation of women with PCOS would be recommended, especially with the methylated form [29,30]. It is known that, in this group, there is a higher probability of women with the adverse methylene tetrahydrofolate reductase (MTHFR) polymorphism [30].

Contrary to expectations, the concentrations of the two remaining vitamins (thiamine and folates) in the plasma did not increase. Thiamine plays a key role in metabolism because it is a cofactor in the transformation reactions of carbohydrates, fats, and amino acids with a branched chain [31]. Due to the disorders of fat and carbohydrate metabolism in women with PCOS, thiamine deficiency supports the development of type 2 diabetes, cardiovascular diseases and dyslipidaemia in patients with PCOS [32]. Other authors have also observed that thiamine level is inversely related to the level of glucose and that it is an important factor in the prevention of the adverse processes of glycation with the production of advanced glycation end products (AGE) [33]. It has also been observed that hyperglycaemia and oxidative stress accelerate the formation of AGE [34]. Furthermore, thiamine supplied with food is a compound that is soluble in water, which makes it more difficult to absorb and it is rather quickly removed from the body through kidneys. This is why it is recommended to supply PCOS patients with benfotiamine, similarly to patients suffering from diabetes [35]. Benfotiamine is a synthetic derivative of thiamine, soluble in fats, that eventually becomes an active form of vitamin B1—thiamine diphosphate, which participates in tissue enzymatic systems of metabolic processes. Thiamine deficiency is associated with the presence of diabetic neuropathy. Diabetic neuropathy is the damage of peripheral nerves in women with PCOS in the early stages before diabetes. The proof of this is the observed elevated level of nerve acid in women with PCOS, also in this particular study group [36]. Women with PCOS and carbohydrate metabolism disorders are often treated with metformin that normalises glycaemia. Its chronic intake is additionally associated with the deficiency of thiamine and cobalamin [7,37]. This is why one of the ideas promoted by the authors of this article is to include supplementation with thiamine and/or benfotiamine while remembering that proper bioavailability in tissues requires the application of high doses [38]. The potential activation of transketolase through benfotiamine contributes to the inhibition of 3 out of 4 mechanisms that damage blood vessels, reducing the risk of cardiovascular disease (CVD) [39]. Another CVD factor in PCOS is the frequently existing elevated level of homocysteine in the plasma [40]. Furthermore, in women with PCOS, the level of homocysteine is inversely correlated with the level of transporting protein (SHBG), with circulatory system diseases and infertility [40]. In order to reduce the level of homocysteine, the triplet of vitamins—B6, B9 and B12 is supplied, and it is worth highlighting that folic acid has the highest influence on the normalisation of its level. Other studies have shown that the synergistic effect of myo-inositol, L-tyrosine, selenium and chromium after 6 months of use restores proper menstruation cycle and ovulation, and it also reduces the body mass of these patients [41,42]. Inositol was introduced as a new agent sensitising towards insulin and androgens in the treatment of patients with PCOS. Contrary to metformin, it does not cause any side effects [43,44]. Furthermore, L-methylfolate increases peripheral sensitivity to insulin, maintaining stable folatemia, thus restoring the normal level of homocysteine. Contrary to folic acid, L-methylfolate has higher bioavailability, no drug/food interference and high absorption level and is stable with reference to the effect of UV-A rays [45]. The supplementation with MI and folic acid has a positive influence on metabolic parameters, especially insulin resistance and the cardiovascular profile in women after 30 years of age, suffering from PCOS [46]. Supplementation with 5 mg of folate every day resulted in the reduction in Hcy in the HOMA-B plasma and a reduction in the concentration of high-sensitivity C-reactive protein (hs-CRP) and in malondialdehyde (MDA) in blood in comparison to folic-1 acid and placebo groups. Moreover, a significant increase in the total antioxidant capacity (TAC) in the plasma and glutathione levels (GSH) was also observed [47].

## 5. Conclusions

To summarise, it is necessary to include antioxidants in the diet of women with PCOS. A proper balanced reduction diet with low GI supplements the level of vitamins soluble in water. However, it is also recommended to include additional supplementation with thiamine (in the form of benfotiamine), niacinamide and folates with inositol, which increase peripheral sensitivity to insulin. The level of vitamin C in the plasma may not be a good marker for its supply in the PCOS group.

## Figures and Tables

**Table 1:** The characteristics of the study group and the control group.

Parameter	PCOS Patients	Control Group (CG)	*p*
Age (year)	32.52 ± 7.12	30.23 ± 6.31	NS
Height (m)	1.67 ± 0.06	1.68 ± 0.06	NS
Body mass (kg)	82.75 ± 15.6	62.76 ± 6.67	1 × 10^−6^
BMI (kg/m^2^)	29.65 ± 6.76	22.22 ± 1.52	1 × 10^−6^
Fat mass (%)	39.54 ± 8.08	25.65 ± 3.96	1 × 10^−6^
Waist circumference (cm)	99.87 ± 15.65	74.75 ± 5.01	1 × 10^−6^
Hip circumference (cm)	109.45 ± 8.96	95.85 ± 4.88	1 × 10^−6^
WHR	0.92 ± 0.08	0.78 ± 0.03 *	1 × 10^−6^

WHR—waist-to-height ratio; BMI—body mass index; *—it does not seem necessary to apply WHR in patients with correct body mass; NS—no statistically significant differences.

**Table 2:** The comparison of the consumption of vitamins with reference to the analysed groups (PCOS-I, PCOS-II, and CG).

Vitamins	PCOS-I *N* = 40	PCOS-II *N* = 18	CG *N* = 15	P PCOS-I vs. PCOS-II	P PCOS-I vs. CG	P PCOS-II vs. CG
C (mg)	68.53 ± 38.22	234.61 ± 87.3	101.37 ± 73.04	1 × 10^−6^	0.017	1 × 10^−5^
B1 (mg)	1.21 ± 0.33	1.64 ± 0.29	1.12 ± 0.32	1 × 10^−6^	0.195	1 × 10^−5^
B2 (mg)	1.33 ± 0.29	1.76 ± 0.59	1.41 ± 0.39	0.0004	0.564	0.041
niacin (mg)	15.42 ± 4.18	21.2 ± 4.65	15.06 ± 4.58	1 × 10^−6^	0.771	0.0001
B6 (mg)	2.08 ± 0.63	2.64 ± 0.78	2.16 ± 0.91	0.002	0.718	0.049
Folates (μg)	221.2 ± 65.4	321.3 ± 72.3	262.8 ± 78.56	1 × 10^−5^	0.041	0.074 *
B12 (μg)	3.30 ± 2.13	3.72 ± 1.05	3.03 ± 2.34	0.323	0.651	0.168

PCOS I—polycystic ovary syndrome group before dietary intervention; PCOS II—polycystic ovary syndrome group after dietary intervention; CG—control group; *—trend.

**Table 3:** The average concentration of vitamins analysed in the plasma before and after the dietary intervention with reference to the analysed groups (PCOS-I, PCOS-II, and CG).

Vitamin [µg/mL]	PCOS-I *N* = 40	PCOS-II *N* = 18	CG *N* = 15	P PCOS-I vs. PCOS-II	P PCOS-I vs. CG	P PCOS-II vs. CG
C	1.032 ± 1.236	1.006 ± 0.581	0.667 ± 0.115	0.287	0.043	0.050
B1	0.256 ± 0.275	0.336 ± 0.282	0.560 ± 0.416	0.241	0.023	0.095 *
B2	0.004 ± 0.002	0.005 ± 0.002	0.006 ± 0.004	0.279	0.112	0.428
B3—nicotinic acid	0.496 ± 0.449	0.153 ± 0.367	0.062 ± 0.050	0.018	0.001	0.370
PP—niacinamide	0.721 ± 0.212	0.756 ± 0.163	0.849 ± 0.201	0.418	0.173	0.292
B5	0.479 ± 0.230	0.606 ± 0.191	0.722 ± 0.192	0.126	0.002	0.116
B6	0.568 ± 0.283	0.758 ± 0.240	0.809 ± 0.217	0.046	0.042	0.770
B7	0.253 ± 0.321	0.262 ± 0.169	0.264 ± 0.256	0.189	0.674	0.419
B9	2.016 ± 0.465	2.130 ± 0.441	2.480 ± 0.616	0.385	0.035	0.083 *
B12	0.056 ± 0.029	0.072 ± 0.073	0.080 ± 0.055	0.386	0.064	0.559

PCOS I—polycystic ovary syndrome group before dietary intervention; PCOS II—polycystic ovary syndrome group after dietary intervention; CG—control group; *—trend.

## Data Availability

Data is contained within the article or Supplementary Material.

## Supplementary Materials

The following are available online at https://www.mdpi.com/2072-6643/13/3/746/s1, Figure S1: The average concentration of vitamins in the plasma with reference to the analysed groups [µg/mL].
