# Supplementary material for: A Properly Balanced Reduction Diet and/or Supplementation Solve the Problem with the Deficiency of These Vitamins Soluble in Water in Patients with PCOS
Source: Nutrients. 2021 Feb 26;13(3):746. doi: 10.3390/nu13030746 (PMC7996738; doi:10.3390/nu13030746)

Figure S1 The average concentration of vitamins in the plasma with reference to the analysed groups [µg/mL]

A. PCOS before and after the dietary intervention (PCOS-I vs PCOS-II)

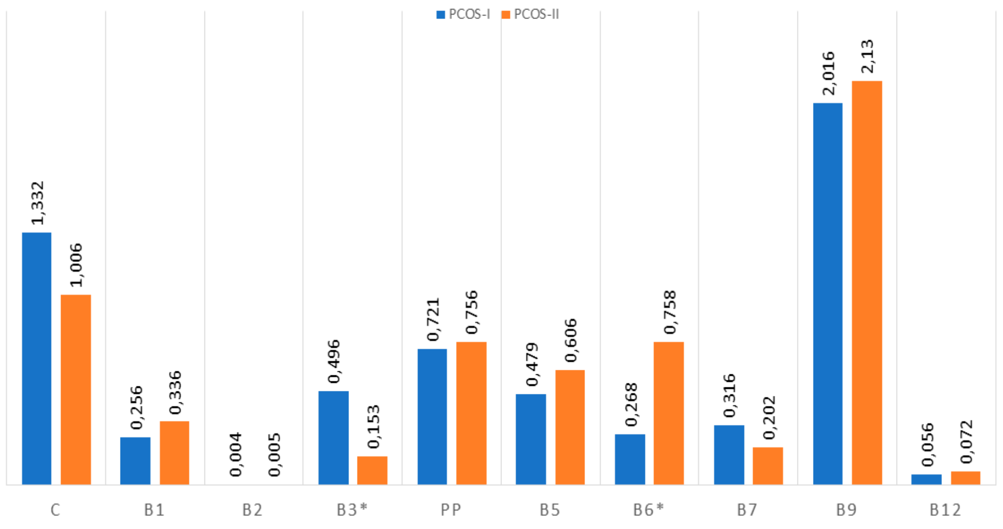

B. PCOS before dietary intervention with reference to the control group (PCOS-I vs CG)

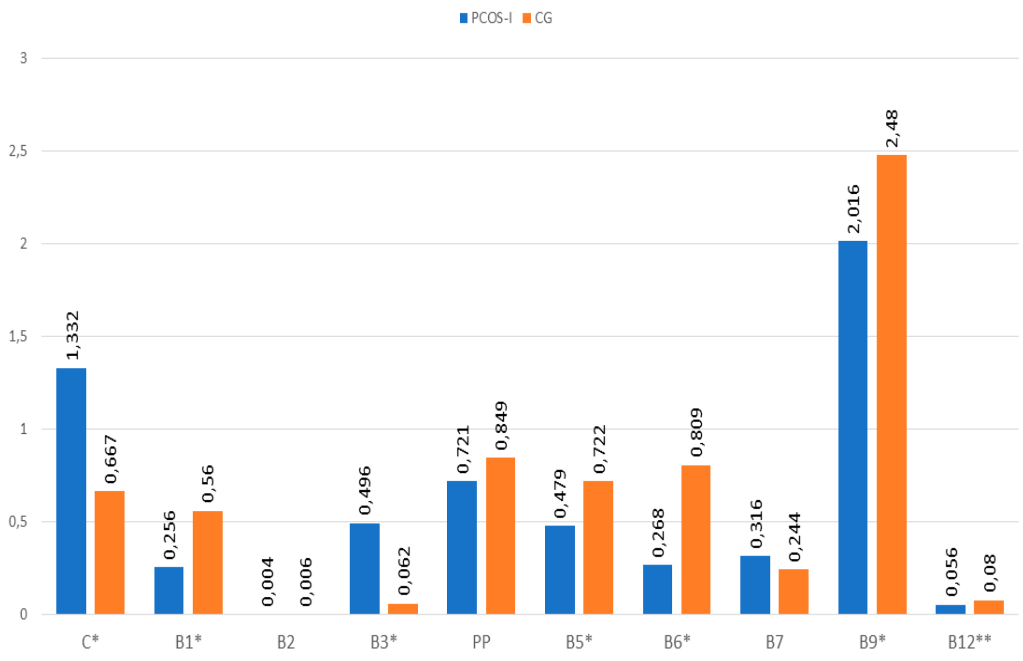

C. PCOS after the dietary intervention with reference to the control group (PCOS-II vs CG)

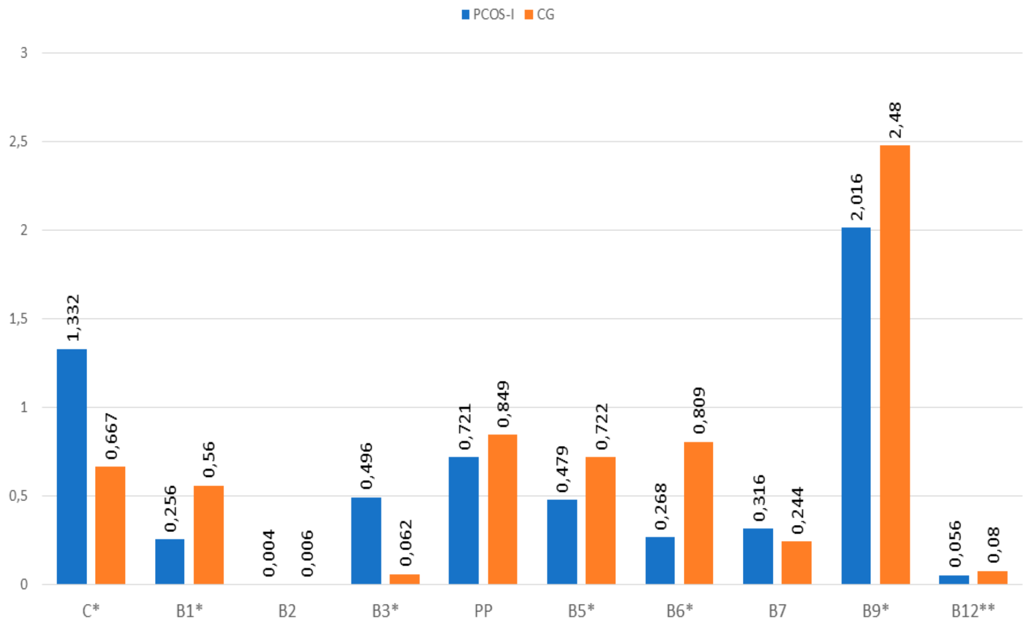

Supplement: Supplementary file 1 [file nutrients-13-00746-s001.pdf]
